# Role of debulking mucoid ACL in unicompartmental knee arthroplasty: a prospective multicentric study

**DOI:** 10.1186/s43019-022-00169-9

**Published:** 2022-10-23

**Authors:** Amyn M. Rajani, Urvil A. Shah, Anmol R. S. Mittal, Sheetal Gupta, Rajesh Garg, Meenakshi Punamiya

**Affiliations:** 1 Breach Candy Hospital, 60 A Bhulabhai Desai Marg, Breach Candy, Cumballa Hill, Mumbai, Maharashtra 400026 India; 2 Surgikids Hospital, 507-509, Aarohi Verve, Nr Vakil Saheb Bridge, Ambli-Bopal Cross Roads, Ambli, Ahmedabad, Gujarat 380058 India; 3 OAKS Clinic, 707 Panchshil Plaza, N S Patkar Marg, opp. Ghanasingh Fine Jewels, next to Dharam Palace, Gamdevi, Mumbai, Maharashtra 400007 India; 4 Galaxy Hospital, Kolar Road, Bhopal, Madhya Pradesh 462042 India; 5 Canadian Hospital, Abu Hail, Dubai, United Arab Emirates

**Keywords:** Mucoid degeneration, ACL, Partial knee replacement, Unicompartmental knee arthroplasty, Debulking, Open debridement

## Abstract

**Background:**

Mucoid degeneration of the anterior cruciate ligament (ACL) has been shown to cause restricted terminal range of motion and rest pain. If present in a patient undergoing unicompartmental knee arthroplasty, it can deteriorate the final outcome. This study aims to compare functional and clinical outcomes of debulking the mucoid ACL in patients undergoing mobile-bearing unicompartmental knee arthroplasty (UKA).

**Methods:**

Patients with mucoid ACL undergoing mobile-bearing UKA at five different centres by five different arthroplasty surgeons were included. They were segregated into two groups matched for all demographic and pre-operative values: group A did not undergo debulking; group B underwent open debulking by a 15-number blade prior to UKA. Patient-related outcome measures, rest pain, clinical outcomes, and subjective patient satisfaction were recorded and compared at 2 years follow-up.

**Results:**

A total of 442 patients (226 patients underwent debulking, 216 patients did not undergo debulking) were included. Both groups showed overall improvement after surgery, however, patients who underwent debulking performed better at 2 years follow-up in terms of Knee Society functional score, International Knee Documentation Committee scores, range of motion, rest pain and overall patient satisfaction (*p* < 0.05) as compared with their counterparts.

**Conclusions:**

Debulking of mucoid ACL in patients undergoing unicompartmental knee arthroplasty significantly reduces the rest pain and improves the final range of motion of the knee joint, subsequently improving the overall functional and clinical outcome of the patient and resulting in greater patient satisfaction.

## Background

Ever since mucoid degeneration of the anterior cruciate ligament (MD-ACL) was named by Kumar et al. just over two decades ago [1], the interest in this condition has quickly gathered pace in the orthopaedic community. Little is known about this condition that affects 1.8–5.3% of the population as per magnetic resonance imaging (MRI) diagnosis [2, 3]. It can be secondary to trauma, or simply degeneration caused by meniscal injury, osteoarthritis or chondral damage [2]. Another theory states that it is a consequence of herniation of a synovial pouch in the substance of the anterior cruciate ligament (ACL), and the subsequent synovial filling inside it; however, the major aetiology of MD-ACL still remains an enigma [4]. The median age of the affected population is estimated to be 51 years [5].

Although asymptomatic in most cases, MD-ACL has been associated with posterior knee pain and restriction of terminal flexion or extension, in addition to pain and discomfort after sitting for a prolonged period of time [4, 5]. This was postulated to be due to the increased tension caused by the mucinous deposition in the substance of the ACL, leading to irritation of the native nociceptors of the tendon and the consequent impingement on the lateral femoral condyle, femoral notch and posterior cruciate ligament (PCL) owing to the increased thickness of the ACL [6–8]. MRI has been established as the gold standard imaging modality for the diagnosis of MD-ACL based on the criteria laid out by Bergin et al. [2].

Unicompartmental knee arthroplasty (UKA) has rapidly emerged as a superior surgical option to total knee arthroplasty for bone-on-bone osteoarthritis owing to less trauma to the surrounding soft tissue, more conservative bone cuts, minimal bleeding, smaller incision, a better range of motion (ROM), faster recovery, preserved kinematics, higher rate of implant survival and decreased chances of major complications such as thromboembolism, cardiac arrest and stroke [9]. However, a predetermined list of criteria has been compiled over the years based on findings in published reports within which a patient needs to fall so as to be eligible to undergo a UKA. These include pristine lateral and patellofemoral compartments, correctable varus deformity, less than 10–15° of fixed flexion deformity, flexion of more than 100°, and functionally intact collateral ligaments and cruciates [10–15].

MD-ACL lies in the grey area of a stable, yet functionally limiting condition that has the propensity to become relatively unstable after undergoing debulking. Despite recent developments suggesting that debulking of MD-ACL does not cause instability [8], the involvement of another surgery that can directly be impacted by it complicates the decision making. When considering a UKA in such a patient, a plethora of dilemmas may arise in the mind of an orthopaedic surgeon vis a vis preserving the MD-ACL at the cost of functional limitation, or debulking it and predisposing the UKA to failure owing to instability.

There is little evidence investigating the effect of MD-ACL in patients undergoing a UKA in the orthopaedic literature. In this study, the authors hypothesised that debulking the MD-ACL with a predetermined, objective endpoint relieves the patient of the rest pain caused by the impingement and improves terminal flexion and functional outcome, without compromising the integrity and stability of the UKA. Hence, study objectives included determining the role and effects of debulking MD-ACL in a patient undergoing UKA as compared with those who do not undergo debulking, by clinical and functional analysis at 2 years post-operatively.

## Methods

### Patient selection and outcome analysis

**Fig. 1:**
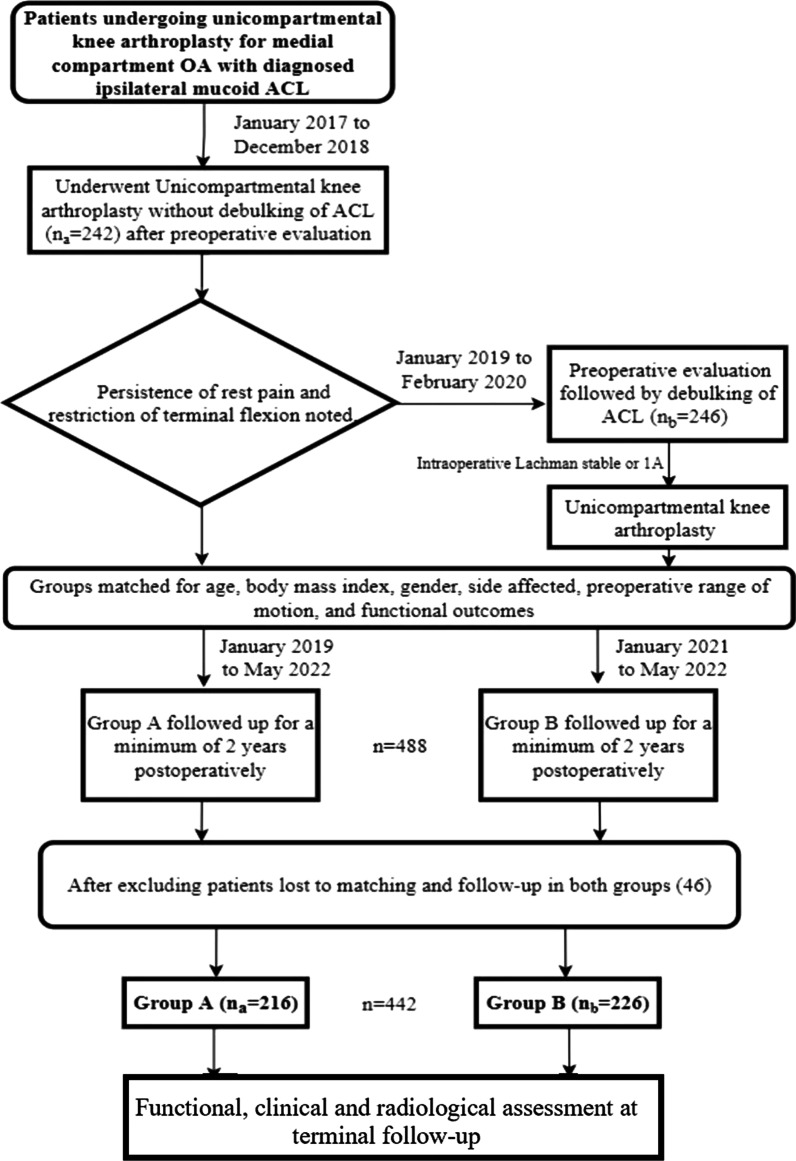
Flowchart representation of selection process of sample size, intervention process and outcome assessment

This was a prospective, cohort, multicentric study conducted in five centres. It included all patients who were diagnosed with mucoid degeneration of ACL and underwent mobile-bearing Oxford partial knee arthroplasty between January 2017 and February 2020, by consecutive sampling method. Informed written consent was obtained from all patients. A total of 488 patients diagnosed with MD-ACL (clinically and radiologically) underwent surgery during this period, of which 46 patients (9.43%) were lost to the minimum 2-year follow-up criteria or to matching of groups. Subsequently, the final sample included 442 patients who were followed-up for at least 2 years after surgery (Fig. 1). The samples collected from the five hospitals were 122 (group A: 58; group B: 64), 68 (group A: 35; group B: 33), 94 (group A: 46; group B: 48), 83 (group A: 41; group B: 42) and 75 (group A: 36; group B: 39), respectively.

Predetermined inclusion and exclusion criteria were used for sample collection. Inclusion criteria was as follows: (1) clinico–radiological confirmation of MD-ACL and medial compartment osteoarthritis (MCOA) of the affected knee joint; (2) patients with less than 10° of varus or 5° of valgus deformity and (3) patients with no flexion contracture. Exclusion criteria was as follows: (1) patients with a previous history of having undergone reconstruction, repair or debulking of the ACL in the ipsilateral knee; (2) patients with a history of any surgery in and around the ipsilateral or contralateral knee, or ipsilateral hip or ankle joints; (3) patients with inflammatory arthritis; (4) patients with musculoskeletal and learning disorders and (5) patients diagnosed with mucoid degeneration of the PCL radiologically.

Pre-operatively, patients were examined and clinico–radiologically confirmed for MD-ACL and medial compartment osteoarthritis (MCOA) of the affected knee. After collecting their demographic details, the ROM of the affected knee was noted, following which they were planned for surgery in the form of mobile-bearing UKA. Pristine condition of the lateral compartment and not more than grade II in the patellofemoral compartment (Ahlbäck classification) was confirmed in all cases. With debulking of MD-ACL being a relatively newer concept and insufficient data backing the eventual stability of the ACL, the surgeons primarily performed UKA without debulking in MD-ACL, until December 2018. However, upon following-up with these patients, and gauging their clinical and functional parameters, complaints of rest pain and restriction of terminal flexion seemed to persist. These patients were included in group A (*n*_a_ = 216). Subsequently, from January 2019, the surgeons started debulking the MD-ACL. They assessed the stability of the joint with the Lachman test intra-operatively, confirming it to be either grade zero or one, with firm end point, before commencing with the UKA [16]. These patients who underwent UKA with debulking of MD-ACL were included in group B (*n*_b_ = 226). Both the groups were matched in terms of age, body mass index, pre-operative ROM, pre-operative International Knee Documentation Committee (IKDC) score [17] and pre-operative Knee Society score (KSS) [18].

The outcome analysis of both groups was done using the same follow-up protocol in terms of time (2 years post-operatively), clinical tests (Lachman’s test), functional scoring systems [IKDC score, KSS, rest pain by visual analogue scale (VAS)] and subjective patient satisfaction. Plain radiographs were taken in anteroposterior and lateral views at 2 years follow-up for radiological analysis in the form of implant failure in all patients or anterior tibial translation in group B by a blinded radiologist. The scores were then compiled and statistically analysed using SPSS-24 software to understand if the debulking resulted in any significant difference in the outcomes.

### Surgical procedure

All the patients were operated on in a leg hanging position and under tourniquet control. By a paramedian incision and mini-midvastus arthrotomy, the joint was exposed. On exposure of the joint, ACL was looked for in terms of integrity and any degenerative changes.

**Fig. 2:**
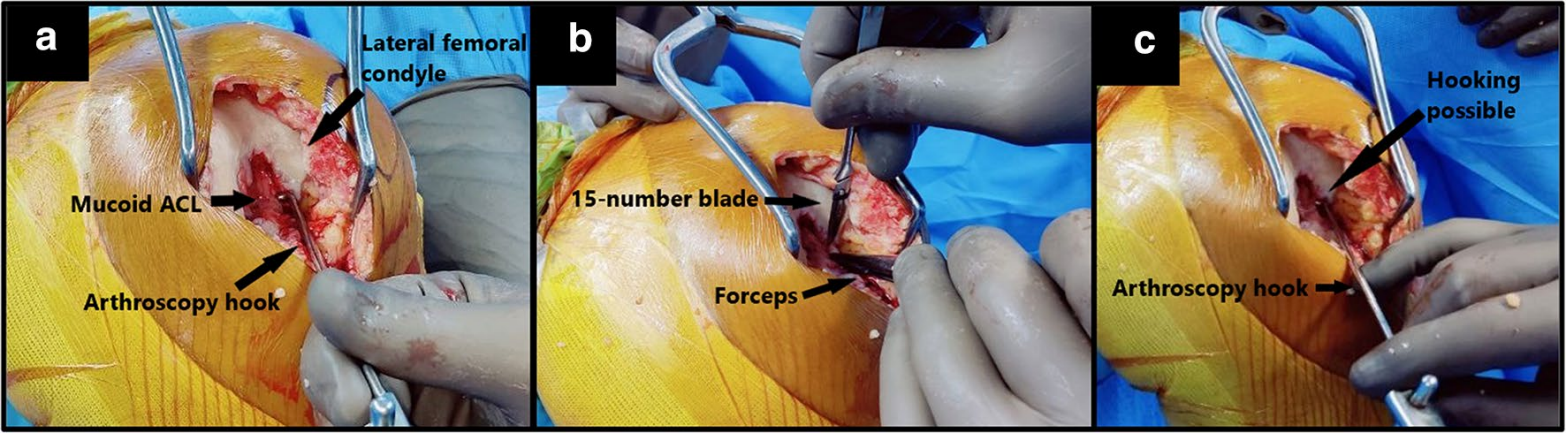
Sequential intra-operative pictures (left to right): **a** Surgeon unable to pass the arthroscopy hook between the medial cortex of lateral femoral condyle and lateral border of mucoid ACL. **b** Open debulking of mucoid ACL under direct vision using 15-number blade. **c** End-point of debulking when the surgeon can pass the arthroscopy hook between the medial cortex of lateral femoral condyle and lateral border of the native ACL effortlessly

On table, the extent of impingement caused by the mucoid degeneration was gauged using an arthroscopy hook. When the arthroscopy hook was attempted to be passed between the lateral femoral condyle and the lateral aspect of the ACL, it was noticed that the impinging fibers of the mucoid ACL blocked its passage, thereby not allowing the hook to be passed between the lateral femoral condyle and the lateral aspect of the ACL, which is otherwise possible in cases with normal ACL. Subsequently, controlled debulking of the posterolateral fibers was done using a 15-number blade until it was possible to tug at the ACL by passing a hook between the lateral femoral condyle and the posterolateral fibres of the ACL (Fig. 2). This denoted adequate release of the impinging fibers against the lateral femoral condyle, femoral notch and PCL. The excised ACL tissue was sent for histopathological confirmation of mucoid degeneration. The rest of the surgery remained the same and all patients underwent mobile-bearing Oxford UKA.

Patients were followed up post-surgery for rehabilitation. At minimum 2 years post-operatively, patients were called for a follow-up, and they were assessed clinically for ROM and stability by a blinded clinician not involved in the clinical care of the patients. Functional assessment was done by the same clinician using patient-related outcome measures (PROMs) using the self-questionnaire-based International Knee Documentation Committee (IKDC) score, Knee Society score (KSS), VAS score (for rest pain) and subjective satisfaction level. All the demographic and biostatistical data was compiled and computed on the SPSS-24 software. A pre-study power analysis was done for a minimum sample size of 400, using dependent and independent variables involved in the study outcome. The power of the study for the corrected model was estimated to be 92% for the aforementioned sample size when keeping the value of *α* as 0.05, the degree of freedom (df) as 1, and adjusted *R*^2^ as 0.023. Hence, we targeted a minimum sample size of 400 patients. Wilcoxon signed rank test was used for longitudinal intra-group comparison of ROM and PROMs between pre-operative and follow-up scores, whereas Mann Whitney *U* test was used to compare ROM and PROMs between the two groups at 2 years follow-up.

## Results

A total of 442 patients (*n*_a_ = 216; *n*_b_ = 226) fulfilled the inclusion criteria and were followed up until a minimum of 2 years post-surgery (mean: 25 ± 2.46 months; range: 24–27.5 months). The demographic data of the study population is presented in Table 1. Histopathological confirmation of mucoid degeneration was obtained in all 442 patients. Grade 1A laxity on Lachman’s test (less than 5 mm translation with a firm end-point) was noted in 27 knees (11.95%) intra-operatively in group B or those who underwent debulking (*n*_b_ = 226), whereas a firm endpoint with no translation was noted in all of the remaining 199 knees (88.05%).

**Table 1:** Descriptive and demographic statistics

	Debulking group (*n*_b_ = 226)	No debulking group (*n*_a_ = 216)	Overall (*n* = 442)	Inter-group distribution comparison*
Age (years)				
Mean	51.947	51.588	51.77	*p* = 0.36
SD	5.666	5.994	5.82	
Gender				
Male	126	121	247	–
Female	100	95	195	–
Side				
Left	112	106	222	–
Right	114	110	220	–
Medial meniscus tear				
Yes	72	59	131	–
No	154	157	311	–
Height (cm)				
Mean	160.22	161.06	160.64	*p* = 0.16
SD	8.46	9.28	8.86	
Weight (kg)				
Mean	68.73	70.19	69.46	*p* = 0.23
SD	6.24	7.87	7.1	
Body mass index (kg/m^2^)				
Mean	26.85	27.07	26.96	*p* = 0.35
SD	3.33	3.68	3.52	

*SD* standard deviation

*(*p* < 0.05 = significant)

On longitudinal, intra-group comparison of the individual groups, it was noted that there was statistically significant (*p* < 0.05) improvement in the ROM of patients who underwent debulking (*Z*-value: 13.047; *p*-value: 0.00) as well as no debulking group (*Z*-value: 9.990; *p*-value: 0.00) at 2 years follow-up, compared with their individual pre-operative ROM. However, the terminal ROM achieved after debulking MD-ACL was significantly better (*p* < 0.05) at 2 years of follow-up as compared with the group that did not undergo debulking. (Table 2).

**Table 2:** Patient-related outcome measures at pre-operative and 2-year follow-up evaluation, and the inter-group comparison

	Debulking group (*n* = 226)		No debulking group (*n* = 216)		Inter-group comparison (Mann–Whitney *U *test)	
	Mean	SD	Mean	SD	*Z*-value	*p*-Value
Knee Society score						
Pre-operative	55.642	3.457	56.335	3.24	1.11	0.1
2-year follow-up	93.60	3.182	87.25	5.488	12.339	**0.000**
IKDC						
Pre-operative	48.26	2.892	48.981	3.17	1.08	0.11
2-year follow-up	82.49	2.649	78.52	4.979	8.86	**0.000**
Flexion (degrees)						
Pre-operative	96.385	5.779	98.09	4.79	2.773	0.06
2-year follow-up	117.89	2.644	103.11	4.096	18.219	**0.000**
Patient satisfaction						
2-year follow-up	9.832	0.375	8.676	0.707	16.083	**0.000**
VAS Score for rest pain						
2-year follow-up	0.13	0.462	2.64	0.954	18.761	**0.000**
Lachman’s test (number of knees)						
Intra-operative	Stable: 199 1A*: 27		–	–	–	–
2-year follow-up	Stable: 201 1A*: 25		–	–	–	–

Boldface indicates statistical significance (*p* < 0.05)

*IKDC* International Knee Documentation Committee, *ROM* range of motion, *VAS* visual analogue scale; *SD* standard deviation

*1A: Less than 5 mm Anteroposterior translation with a firm end point

There was a statistically significant improvement in the mean IKDC and mean KSS of both the groups at 2 years of follow-up as compared with their pre-operative scores (*p* < 0.05). On inter-group comparison, the group in which debulking was done had better mean KSS and mean IKDC score at 2 years of follow-up (*p* < 0.05).

On inter-group comparison, VAS score for rest pain of the debulking group (0.13 ± 0.46) was also found to be lower than the non-debulking group (2.64 ± 0.95) (*p* < 0.05). Finally, the patients who underwent debulking had a better mean subjective patient satisfaction (9.83 ± 0.37 versus 8.68 ± 0.71) at the final follow-up at 2 years (*p* < 0.05) (Table 2).

**Fig. 3:**
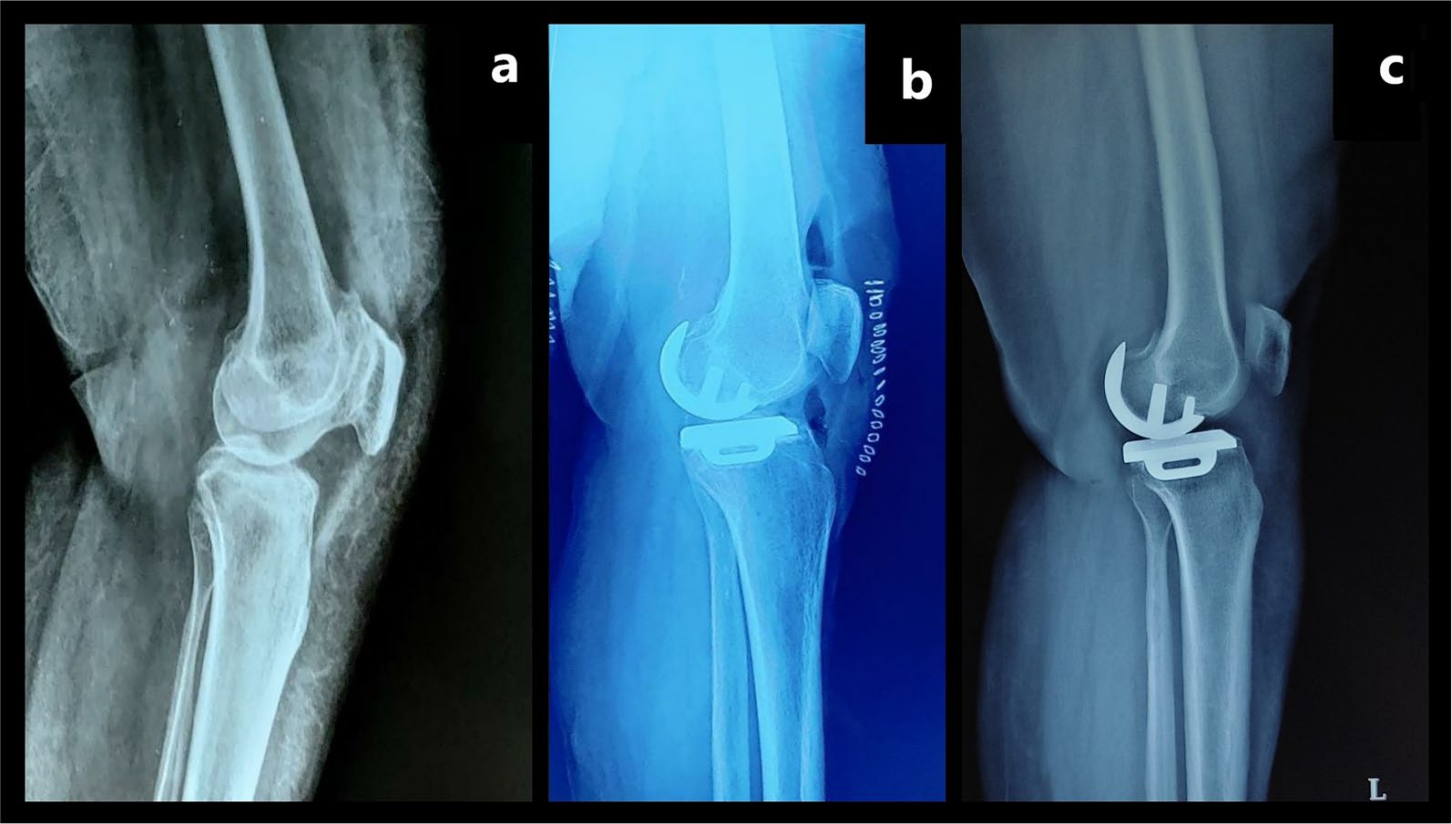
Plain radiographs in lateral view (in standing) of the left knee of patient number 164 from group B (Left to Right): **a** Pre-operative radiograph for baseline position of posterior femur and tibia. **b** Immediate post-operative radiograph showing no posterior translation of femur compared with pre-operative radiograph. **c** 24.5 months post-operative radiograph showing no posterior translation of femur compared with pre-operative and immediate post-operative radiograph

Plain radiographs of all the patients taken in anteroposterior and lateral views (in standing) at 2 years follow-up showed no signs of implant failure in any patient. Pre-operative and follow-up lateral radiographs of the patients who underwent debulking were analysed by a blinded radiologist and no posterior translation of the femur was noted, thereby confirming no instability caused by the debulking (Fig. 3).

## Discussion

UKA by itself improves the functional and clinical outcome of patients with medial compartment osteoarthritis. However, the presence of MD-ACL means the associated impingement symptoms have the propensity to continue despite the arthroplasty. In these patients, when UKA is coupled with debulking of the MD-ACL with a predetermined end point, a significant improvement in the overall ROM, rest pain, functional outcome scores and subjective satisfaction of the patient can be achieved.

With the advent of the mobile-bearing UKA system, the predilection towards this surgery is on an upward trend. Despite integrity and status of cruciates being paramount in a UKA, no study has evaluated the effect of debulking MD-ACL in this procedure. Mucoid degeneration of the anterior cruciate ligament is a rare pathological entity with disputed theories of origin [1–4]. It is characterised by infiltration of mucoid-like substance (glycosaminoglycans) interspersed within the substance of ACL leading to thickening of the native ACL and its impingement on the lateral femoral condyle, femoral notch and PCL, consequently causing knee pain and limited motion. It is commonly associated with characteristic clinical findings such as progressive knee pain and discomfort on prolonged sitting or standing in a fixed posture, without history of a significant trauma or instability preceding the symptoms.

This study showed that the patients who underwent debulking had statistically superior knee function, lesser rest pain and greater satisfaction rates at 2 years of follow-up compared with those in whom MD-ACL was not debulked. Various techniques have been proposed for the debulking of MD-ACL including arthroscopic debridement using shaver or radiofrequency ablation, with or without notchplasty, that have shown to give good clinical results [8, 19]. The demographic distribution of study population with respect to the mean age, gender distribution and the side affected was similar to the recent studies done on the MD-ACL as a standalone entity [2, 3, 5, 8]. A few studies have shown direct correlation between the presence of meniscus tear in patients with MD-ACL. As per study population statistics, the prevalence of medial meniscus tears (29.64%) in this study were congruent to the rates found in studies by Srivastava et al. [20], Chudasama et al. [21] and Ventura et al. [22].

Ventura et al. [22], Pandey et al. [6] and Lintz et al. [4] showed high percentages of laxity post debulking of MD-ACL, a finding which would not allow a surgeon to carry out a UKA after the debulking. However, all of these debridements were done under arthroscopy, whereas the surgeons in this study progressively debulked the ACL by open method using a 15-number blade, under direct vision. The end point was ascertained to be when the surgeon could pass a hook between the medial cortex of the lateral femoral condyle and the lateral border of the remaining ACL. Lachman’s test was performed intra-operatively in the immediate aftermath of the debulking, and only 27 (11.95%) knees were found to have grade one laxity with a firm end point, while no laxity was noted in the other knees. These findings were in line with the findings published by Cha et al. [5]. The authors presume that this objective end point suggests adequate debulking, just enough to remove the impingement factor from the lateral femoral condyle, femoral notch and PCL, and consequently the symptoms associated with the impingement. This was confirmed at 2 years follow-up, when the patients who underwent debulking showed a statistically significant improvement in in mean terminal flexion by 21.51° in group B (mean pre-operative: 96.385° ± 5.78°; mean post-operative: 117.89° ± 2.64°) as compared with 5.02° in group A (mean pre-operative: 98.093 ± 4.79°; mean post-operative: 103.11° ± 4.1°). Although a few studies such as the one by Lee et al. [7] have documented the presence of flexion contracture, we did not notice the same in any patient from the recruited sample size. The follow-up period was similar to that of Cha et al. [5] and Chudasama et al. [21]. The second most common complaint in patients with MD-ACL, namely rest pain, also showed statistically significant reduction at 2 years follow-up in patients who underwent debulking as compared with their counterparts, as measured by VAS score.

Corroborating these findings were the improvements and the final PROMs of both the groups which corresponded with the findings of the literature review by Sweed et al. on nine studies [8]. While both the groups showed improvements in the functional scores post-surgery, which could be attributed to the UKA primarily, the finding that patients in whom debulking was done had better mean IKDC and mean KSS at 2 years follow-up confirmed the study hypothesis that debulking improves the long-term outcome in patients with MD-ACL undergoing UKA. Culmination of these results meant that even the patient satisfaction at 2 years follow-up was better in the debulking group. Hence, the improvement in ROM, attenuation of rest pain and better long term functional outcome measures supported the hypothesis regarding the positive effect of debulking a MD-ACL in patients undergoing UKA.

This study holds clinical significance in terms of the very limited literature available on the role of MD-ACL in outcomes of patients undergoing UKA. It provides objective end points for open debulking of mucoid ACL in these patients and, being a multicentric study, it entails external validity of this procedure in a diverse population. With an increasing trend of UKA being performed worldwide, the role of addressing concomitant MD-ACL is vital in improving the overall outcome of the patient without causing any instability or implant failure, as shown in this study.

The authors acknowledge some limitations to this study. Only mobile-bearing UKA design was used in all the patients; hence the findings may differ in patients undergoing UKA with different implant designs such as fixed-bearing UKAs. Secondly, all the patients underwent the surgery and postoperative rehabilitation based on a standardized protocol which may limit the reproducibility of results if not adhered to. Both the patient groups were matched for age, gender, pre-operative ROM and functional scores, minimising any confounding effect in data collection. Despite maximising the factors involved between the two groups, as the sample size collection was done during two separate time periods and consecutive sampling was done instead of randomisation, there is a possibility of sampling bias. Being a multicentric study involving five different surgeons, surgeon factors could potentially affect surgical procedure and clinical outcome, despite a predetermined endpoint for the debulking. The authors did not perform stress tests. The stability measurements were subjective, and although undertaken by a blinded clinician, could be susceptible to human error. They were however, supplemented by other objective methods such as comparison of pre-operative, post-operative and follow-up status of lateral view of plain radiograph in standing position, to look for any posterior translation of the femur which would suggest instability. Considering a dearth of studies that evaluates the effect of debulking an ACL with mucoid degeneration, more prospective studies with larger sample size and longer follow-up period are encouraged to corroborate the findings of this study.

## Conclusion

In patients undergoing UKA and diagnosed with mucoid degeneration of the ACL, controlled debulking of the ACL provides better functional outcomes as compared with non-debulking of the mucoid ACL. Stability of the joint is not compromised on controlled debulking. To summarize, debulking of mucoid ACL in patients undergoing unicompartmental knee arthroplasty significantly reduces the rest pain and improves the final range of motion of the knee joint, subsequently improving the overall functional and clinical outcome of the patient and resulting in greater patient satisfaction.

## Data Availability

The datasets used and/or analysed during the current study are available from the corresponding author on reasonable request.

## Abbreviations

MD-ACL: Mucoid degeneration of the anterior cruciate ligament
MRI: Magnetic resonance imaging
ACL: Anterior cruciate ligament
PCL: Posterior cruciate ligament
UKA: Unicompartmental knee arthroplasty
ROM: Range of motion
MCOA: Medial compartment osteoarthritis
IKDC: International Knee Documentation Committee
KSS: Knee Society score
VAS: Visual analogue scale
PROMs: Patient-related outcome measures
